# Role of Sulphur and Heavier Chalcogens on the Antioxidant Power and Bioactivity of Natural Phenolic Compounds

**DOI:** 10.3390/biom12010090

**Published:** 2022-01-06

**Authors:** Maria Laura Alfieri, Lucia Panzella, Riccardo Amorati, Alice Cariola, Luca Valgimigli, Alessandra Napolitano

**Affiliations:** 1 Department of Chemical Sciences, University of Naples “Federico II”, Via Cintia 21, I-80126 Naples, Italy; marialaura.alfieri@unina.it (M.L.A.); panzella@unina.it (L.P.); 2 Department of Chemistry “Giacomo Ciamician”, University of Bologna, Via S. Giacomo 11, I-40126 Bologna, Italy; riccardo.amorati@unibo.it (R.A.); alice.cariola2@unibo.it (A.C.)

**Keywords:** Bond Dissociation Energy, kinetic constant, DFT calculations, thia/selenotocopherols, S/Se/Te-substituted phenols, lipid peroxidation, alkylperoxyl radicals, antioxidant chemical assays, glutathione peroxidase-like activity, glutathionyl/cysteinylcatechols, dihydrolipoic acid

## Abstract

The activity of natural phenols is primarily associated to their antioxidant potential, but is ultimately expressed in a variety of biological effects. Molecular scaffold manipulation of this large variety of compounds is a currently pursued approach to boost or modulate their properties. Insertion of S/Se/Te containing substituents on phenols may increase/decrease their H-donor/acceptor ability by electronic and stereo-electronic effects related to the site of substitution and geometrical constrains. Oxygen to sulphur/selenium isosteric replacement in resveratrol or ferulic acid leads to an increase in the radical scavenging activity with respect to the parent phenol. Several chalcogen-substituted phenols inspired by Vitamin E and flavonoids have been prepared, which in some cases prove to be chain-breaking antioxidants, far better than the natural counterparts. Conjugation of catechols with biological thiols (cysteine, glutathione, dihydrolipoic acid) is easily achieved by addition to the corresponding *ortho*-quinones. Noticeable examples of compounds with potentiated antioxidant activities are the human metabolite 5-*S*-cysteinyldopa, with high iron-induced lipid peroxidation inhibitory activity, due to strong iron (III) binding, 5-*S*-glutathionylpiceatannol a most effective inhibitor of nitrosation processes, and 5-*S*-lipoylhydroxytyrosol, and its polysulfides that proved valuable oxidative-stress protective agents in various cellular models. Different methodologies have been used for evaluation of the antioxidant power of these compounds against the parent compounds. These include kinetics of inhibition of lipid peroxidation alkylperoxyl radicals, common chemical assays of radical scavenging, inhibition of the OH• mediated hydroxylation/oxidation of model systems, ferric- or copper-reducing power, scavenging of nitrosating species. In addition, computational methods allowed researchers to determine the Bond Dissociation Enthalpy values of the OH groups of chalcogen modified phenolics and predict the best performing derivative. Finally, the activity of Se and Te containing compounds as mimic of glutathione peroxidase has been evaluated, together with other biological activities including anticancer action and (neuro)protective effects in various cellular models. These and other achievements are discussed and rationalized to guide future development in the field.

## 1. Natural Phenolic Compounds as a Valuable Source of Molecular Scaffolds Endowed with Diverse Biological Activities

The variety of molecular scaffolds offered by phenolic compounds occurring in nature is huge as is the variety of roles that they can play in the organisms in which they are produced. Even more interesting are the biological activities that may be exerted in humans, which, coupled with their occurrence in easily accessible sources, have attracted in the recent literature, an enormous interest toward these classes of compounds. Most of these studies have highlighted how the activity of polyphenols is primarily associated to their antioxidant potential, but it is ultimately expressed in a variety of biological effects. For recent reviews see [1,2,3,4,5].

Manipulation of the molecular scaffold of natural phenols that could improve the biological activity without affecting or with acceptable alterations of the biocompatibility is nowadays a largely pursued approach to access rather complex molecular systems by relatively simple synthetic procedures.

Examples of how chemical transformations of natural phenols may lead to the enhancement, suppression, or modification of their biological activities as a result of changes in the chemico-physical properties, including solubility, partition in the biological compartments or, more specifically, changes in receptor recognition, have been collected in several reviews [6,7,8,9]. Semisynthetic derivatives of polyphenols have also been largely prepared with the aim to overcome the limited bioavailability and low concentrations at the target tissues of these compounds, thereby gaining improved pharmacological and pharmacokinetic properties through functionalization. An extensive review article has appeared in the present special issue that offers an overview of the functionalization of the hydroxy groups in phenols through acylation or esterification/amidation of the carboxy groups in phenolic acids, so to modify lipophilicity or to install moieties endowed with a biological activity *per se* [10].

In this context, of particular interest is the functionalization of natural phenols that mimic those occurring in metabolic pathways in living organisms.

A number of semisynthetic gallates, e.g., galloylated resveratrol and others, have been prepared, which showed enhanced cytotoxicity and anticancer activities [11]. Different chemical and enzymatic sulfation protocols have been developed in the light of the variety of properties that sulfation imparts to the polyphenol functionalities [12,13,14,15]. Methylation and glucuronidation are metabolic strategies to impart OH functionalities of metabolites/xenobiotics higher solubility in water or to inhibit their oxidative transformations [16,17].

This is also the case of conjugation with biological thiols, primarily glutathione (GSH) and cysteine, a modification that has been regarded mostly as a way of improving solubility in physiological fluids of endogenous and exogenous compounds, favoring their excretion, but much less as a strategy to modify their redox properties and ultimately their biological activities.

The present review is framed in this context, which focuses on specific modifications of phenols/polyphenols by insertion of S/Se/Te-containing functionalities on the molecular scaffold or on replacement of oxygen atoms with heavier chalcogens. The effects of these modification on the activity of the phenolic compounds, with particular reference to the antioxidant properties, are also presented, as evaluated by conventional chemical assays [18] and different model systems in association with computational approaches. These studies, which will be illustrated in the following review, contribute to and draw a well-defined picture of the mode of action of polyphenols and how their activity is affected by chalcogens from the viewpoint of the chemical mechanisms, an issue that is not frequently addressed in discussions regarding the activity of natural polyphenols.

## 2. Electronic and Stereo-Electronic Effect and H-Bonding of Chalcogens as Substituents in the Phenolic Ring of Simple Molecules

**Scheme 1:**
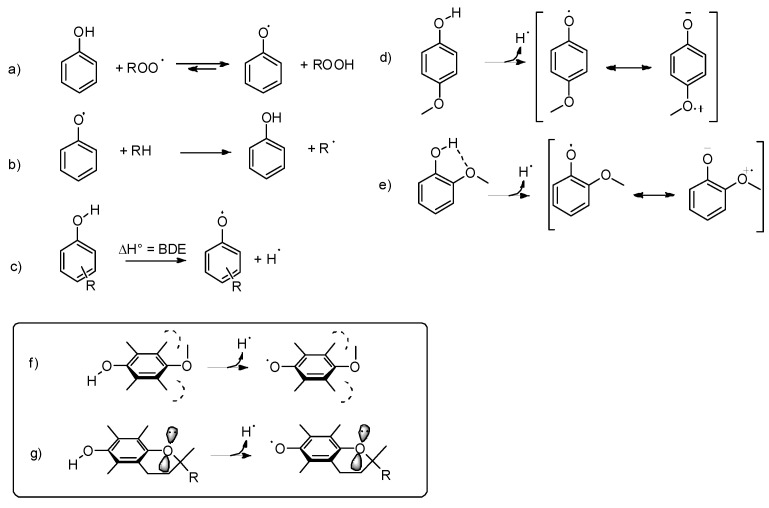
H-donation, electronic and intramolecular H-bond effects in substituted phenols (**a**–**g**).

The antioxidant activity of phenols stems mainly from their ability to donate a H-atom to alkylperoxyl radicals (ROO•, see Scheme 1a), without contributing to propagate the peroxidative radical chain by reacting with the substrate (Scheme 1b) or with hydroperoxides (Scheme 1a). These properties are inversely related to the Bond Dissociation Enthalpy (BDE) of the phenolic OH, which in turn depends on the substituents present on the aromatic ring (Scheme 1c). Electron-withdrawing (EW) substituents increase the BDE (OH) mainly by stabilizing the phenol. In contrast, electron-donating (ED) substituents, such as alkyl and methoxy groups, in position *ortho* and *para* to the phenolic OH lower the BDE (OH) by charge donation to the phenoxyl radical (which is an EW group); therefore, they enhance the antioxidant activity [19]. However, when H-bond accepting substituents, such as methoxy groups, are present in *ortho* position to the phenolic OH, they have a BDE-increasing effect that may counterbalance their aforementioned BDE-lowering effect. For example, the BDE (OH) of phenol is 86.7 kcal/mol [20], that of 4-methoxyphenol is 81.6 kcal/mol, while that of 2-methoxyphenol is 84.9 kcal/mol (see Scheme 1d,e) [21]. Moreover, too many or too strong ED groups on a phenol also lower its oxidation potential, rendering it too reactive toward O_2_, thus reducing its stability under air and causing a pro-oxidant behavior [22].

The geometrical constraints required for a good electronic interaction between the OH/O• group and the substituents give rise to a series of stereo-electronic effects that have to be taken into account to rationalize the antioxidant activity of phenols. For instance, if a *para* methoxy group is forced to a perpendicular orientation by the presence of two methyl groups in *meta* position, its effect on the BDE (OH) is almost negligible, because the stabilization of the phenoxyl radical requires that an oxygen lone pair interacts with the aromatic π system. On the other hand, if a *para*-OR substituent is forced to a nearly planar conformation by the inclusion into a cycle, it stabilizes the radical irrespectively of the *meta* substituents as the conjugation of its heteroatom lone pair is forced by geometrical constrains, as observed, for instance, in natural tocopherols (Scheme 1f,g) [23].

**Scheme 2:**
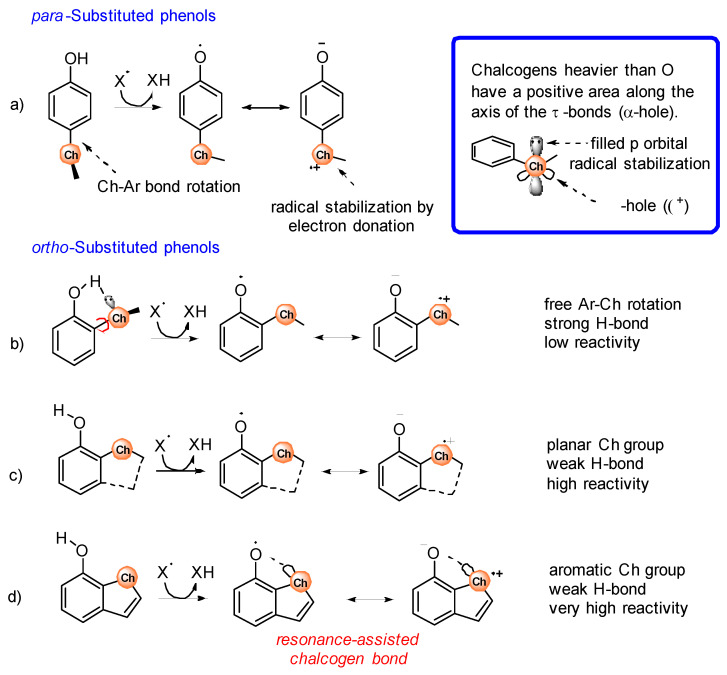
Stereo-electronic effects of chalcogen-containing substituents, *in para* (**a**) or *ortho* (**b**–**d**) positions, on the reactivity of phenols with free radicals (X•).

Substituents containing heavy chalcogens (ChR, see Scheme 2) directly attached to the aromatic ring have peculiar effects on the reactivity of phenols with peroxyl radicals (ROO•) that differ from those of alkoxy (OR) substituents, although chalcogens are commonly assimilated to oxygen. A typical characteristic of heavy chalcogens (Ch) is the presence, on their surface, of a region of positive charge located on the outer side of σ-bonds, named σ-hole (see inset in Scheme 2). The dimension of the σ-hole increases with the Ch size (S < Se < Te) and when EW substituents are attached to Ch. The interaction of a Ch σ-hole with a lone pair of another atom is responsible for the formation of the so-called *chalcogen bonds*, which are noncovalent interactions that have been recently found to play an important role in conformational preferences and molecular recognition [24,25].

ChR substituents in *para* position in phenols adopt a perpendicular geometry to provide an interaction between the electron-poor σ-hole and the electron-rich π-system (see Scheme 2a) [26]. When the ChR substituent is in *ortho*-position to the phenolic OH, the perpendicular conformation of the ChR group is further stabilized by the H-bond interaction of the filled p-orbital of the Ch atom with the OH group (see Scheme 2b) [26]. Interestingly, if the *ortho*-ChR group is forced in a planar conformation by inclusion in a 5 or 6-membered cycle, the H-bond is not formed because of the wrong orientation of the filled p-orbital (Scheme 2c,d) [27].

The stereo-electronic effects described above strongly influence the reactivity of the Ch-substituted phenols with alkylperoxyl radicals ROO• involved in lipid peroxidation and with free radicals, in general (herein, indicated with X•). During the H-atom transfer from the phenol to X• the aromatic ring develops a significant spin density, and the phenoxyl oxygen becomes electron poor, so the first and more obvious effect of ChR is that of stabilizing the phenoxyl radical by electron donation by sharing the filled p orbital (Scheme 2a). However, this is not possible when the ChR is in the perpendicular conformation. Phenols having an *ortho*-ChR substituent free to rotate about the Ar-ChR bond react very slowly with radicals because of the presence of the intramolecular H-bond that stabilizes the phenol, and because of the wrong arrangement of the ChR group, that interacts with the aromatic π-system of the incipient radical using the σ-hole instead of the filled p-orbital [28]. In the case of *para* substituted ChR phenols, this effect is less evident because the preference for the perpendicular conformation is less important than in *ortho*-ChR phenols [26]. Of course, if the *ortho*-ChR substituent is forced to be planar by inclusion into a ring, the interaction of the filled p orbital is possible, leading to good stabilization of the radical and fast reaction with X• (Scheme 2c) [27]. A specific effect is observed when the *ortho*-ChR group is included into an aromatic cycle, such as in 7-OH benzothiophene (Scheme 2d). The combined EW effect of the aromatic ring and of the phenoxyl oxygen causes the formation of a through-space interaction between the ChR and the ArO• groups with characteristics similar to a chalcogen bond that further stabilizes the radical, leading to a very fast reaction with free radicals [29].

**Scheme 3:**
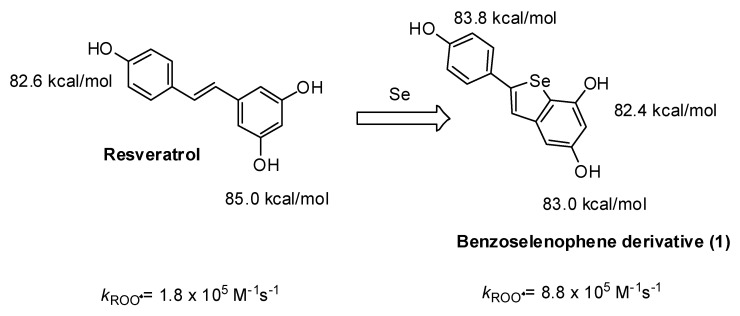
Effect of the insertion of a selenium atom into the molecular scaffold of resveratrol on the BDE of the OH groups and on the rate constant of the reaction with ROO• radicals.

An interesting application of these principles is represented by a semisynthetic phenolic antioxidant derived from *trans*-resveratrol reported in Scheme 3. The resveratrol-derived benzoselenophene (1) reacted 5 times faster than the parent natural phenol, owing to the stabilization of the radical due to the Se-atom [30]. Interestingly, also the bioactivity of this derivative was significantly changed with respect to the parent resveratrol. The benzoselenophene derivative exerted a higher protective effect than resveratrol against oxidative stress in intestinal myofibroblasts and osteocytes [31]. Moreover, the selenylated compound was able to inhibit human carbonic anhydrases, with Ki values in the range 5–60 μM [32].

**Figure 1:**
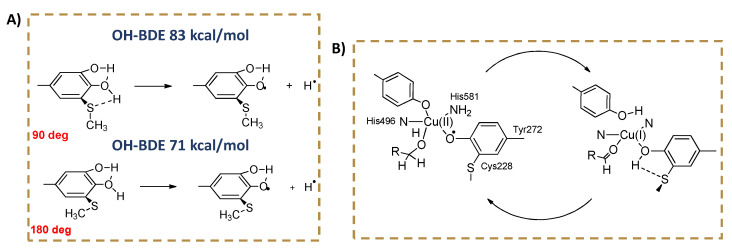
(**A**) Stereo-electronic effects of a hypothetical *ortho*-SCH_3_ substituent on the BDE of a phenol. In red are shown the Ar-SMe dihedral angles. (**B**) Active site of the enzyme Galactose Oxidase, featuring a covalent Tyr-Cys linkage.

Because of these stereo-electronic effects, it has been shown by theoretical calculations that an *ortho*-alkylthio substituent, forced to adopt the same geometry in the phenol and in the radical, has dramatically different effects on the phenolic BDE (OH) depending on the dihedral angle it forms with the aromatic ring (Figure 1), varying from EW to ED [33]. These effects may become important in a protein or in a membrane surface, where free rotation around the Ar−S bond could be impaired by local constrains. It has been proposed that the stereo-electronic effect of the alkylthio substituent may play a role in promoting the substrate oxidation in the active site of the radical enzyme galactose oxidase, featuring a tyrosine covalently linked to a cysteine in *ortho* position with respect to the reactive OH (Figure 1) [34].

## 3. Isosteric O to S or Se Replacement

**Figure 2:**
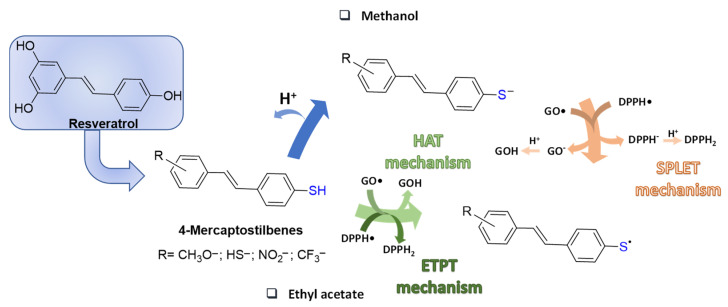
Resveratrol-related 4-mercaptostilbenes and their radical scavenging mechanisms.

Replacement of oxygen with sulphur in the scaffold of naturally occurring phenols has been pursued as a strategy to prepare more effective antioxidants, which might also exhibit a potentiation of the biological activity. A representative case is that of resveratrol for which substitution of a 4′-SH for the 4′-OH group in the stilbene scaffold was systematically investigated in a series of resveratrol-related 4-mercaptostilbenes (Figure 2). The hypothesis that thiophenol should be a stronger radical scavenger than phenol was assessed by measurement of the kinetics of reaction with galvinoxyl (GO•) and 2,2-diphenyl-1-picrylhydrazyl (DPPH•) radicals in different solvents. Indeed, some of the 4-mercaptostilbenes synthesized proved up to 10^4^ times more active than resveratrol in methanol, and a hundred fold more effective than known antioxidants (α-tocopherol, ascorbic acid, quercetin, and trolox). The mechanism of interaction with the radicals was detailed by kinetic measurements and found to be dependent on the type of solvent. This effect is well beyond the change in reactivity of DPPH• due to the polar solvent [35]. In methanol, a solvent that supports ionization, the reactions proceed mainly through a sequential proton loss electron transfer mechanism (SPLET), while in solvents that weakly supports ionization such as ethyl acetate, a HAT (Hydrogen Atom Transfer) reaction takes place in the case of GO•, or an ETPT (Electron Transfer Proton Transfer) in the case of DPPH• [36] (Figure 2).

The case of isosteric -OH to -SH replacement in ferulic acid is also noteworthy. The differences in the kinetics of the reaction with DPPH•, ABTS•^+^ and GO• of thioferulic acid with respect to ferulic acid were interpreted in terms of the higher acidity of the former and, also in this case, SPLET with the electron transfer from the thiophenolate anion to a radical was considered the preferential mechanism in solvents with higher ability to solvate and, thus, stabilizes anions such as water or alcohols. Interestingly, thioferulic acid exhibited a marked protective effects of neuroblastoma cells against oxidative damage inflicted by both H_2_O_2_ and *tert*-butyl hydroperoxide (*t*-BHP), while ferulic acid was almost ineffective, hinting at these compounds as a new class of neuroprotective agents [37,38].

The higher antioxidant activity of thiophenols as peroxyl radicals’ scavengers with respect to phenols was confirmed also by computational DFT studies on differently substituted thiophenols. Single electron transfer (SET) was found to be the main mechanism, with rate constants close to the diffusion limit, suggesting that these compounds are able to scavenge peroxyl radicals before they can damage biomolecules [39].

**Figure 3:**
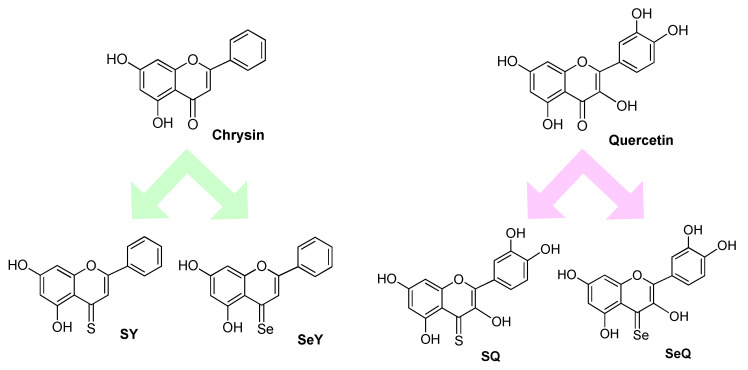
Chemical structures of natural flavonoid (e.g., chrysin and quercetin) and their sulphur and selenium derivatives.

DFT analysis was also used to predict the effects of isosteric substitution of oxygen with sulphur or selenium in two natural well-known antioxidants such as quercetin and Crysin (Figure 3). While in the case of chrysin compounds SY and SeY a significant increase in the hydroperoxyl radical scavenging activity was observed, this was not appreciable in the case of quercetins SQ and SeQ, possibly because the activity of the parent compound is already very high. Kinetic studies showed that for SY the reaction with the peroxyl radicals is 2.4 times higher than that of the parent compound while the selenium derivative of chrysin was predicted to be the most potent member of the family with an overall constant rate more than 4 orders of magnitude larger than that of chrysin itself. This derivative remains very active also in lipid media where the other chrysins lose their activity. All the quercetins (O, S or SeQ) proved to be exceptionally good scavengers of peroxyl radical in aqueous solutions at neutral pHs, while in lipid media, the sulphur derivative SQ proved to be the most efficient one, with rate constants in the order of 3.7 × 10^4^ M^−1^s^−1^. pH of the aqueous solutions also turned out as a critical parameter affecting the rates of the reaction. While for chrysin and its derivatives, the higher the pH the faster the reaction with peroxyl radicals, quercetins are predicted to exhibit their maximum peroxyl radical scavenging activity at pH values from 7 to 10 [40].

## 4. Chalcogen-Substituted Phenolic Antioxidants Inspired by Vitamin E and Flavonoids

**Scheme 4:**
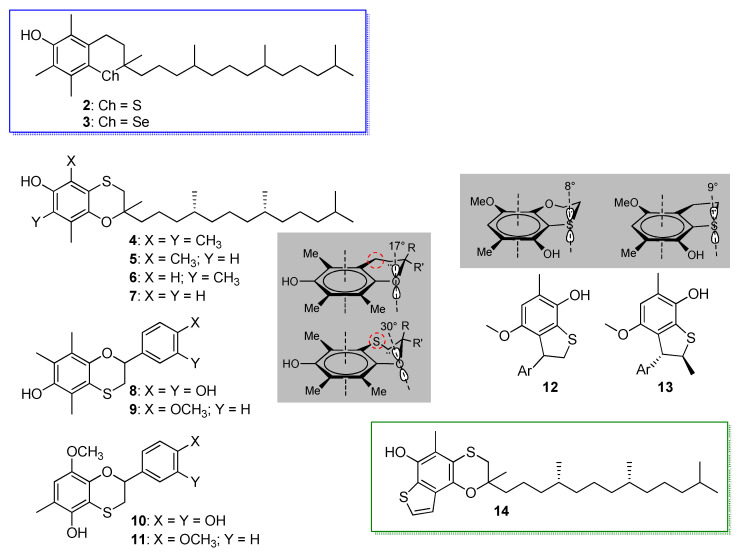
Thiatocopherols, selenotocopherol and related antioxidants modified by insertion of sulphur.

Vitamin E is arguably the most important natural phenolic antioxidant, hence, not surprisingly, it has served as a source of inspiration for the design of novel redox-active molecules [41,42]. Insertion of chalcogens in its core structure has been a long-standing strategy and the 11-steps synthesis of all-rac. α-thiatocopherol (**2**) by Ingold et al. dates back to 1988 [43], while that of all-rac. α-selenotocopherol (**3**) followed 18 years later [23] (Scheme 4). Both compounds had lower reactivity with ROO• radicals than natural α-tocopherol (α-TOH) (*k*_inh_ was 1.3 × 10^6^ M^−1^s^−1^ and 1.2 × 10^6^ M^−1^s^−1^, respectively, vs. 3.2 × 10^6^ M^−1^s^−1^ at 303 K in chlorobenzene) [23,43], owing to the ca. 1 kcal/mol higher BDE (OH) due to the lower ED character of S and Se compared to O [26].

Building on these findings, an alternative approach to insert an endocyclic chalcogen in the chromanol structure as a substituent of the aromatic ring, while maintaining the oxygen, was the replacement of the –CH_2_– in position 4, which afforded the α-,β-,γ- and δ-4-thiatocopherols **4-7** (Scheme 4). All of them proved to be excellent chain-breaking antioxidants, trapping ROO• radicals with a constant rate, which was about 50% that of the natural counterparts [44]. The same synthetic strategy also afforded a family of hydroxy-4-thiaflavanes, among which were compounds **8** and **9** that represented a hybrid among the core structures of tocopherol and catechin [45] (Scheme 4). They trapped ROO• radicals with a rate constant *k*_inh_ of 1.4 × 10^6^ and 1.0 × 10^6^ M^−1^s^−1^, respectively, (at 303 K in chlorobenzene), having higher antioxidant activity than catechol (*k*_inh_ = 5.6 × 10^5^ M^−1^s^−1^), yet, disappointingly, still lower than reference α-tocopherol [46]. The lower than expected reactivity of all the compounds **4**–**9** bearing the same core was explained on the basis of a less favorable stereo-electronics compared to natural tocopherols, caused by the distortion of the fused 6-membered ring imposed by the presence of sulphur. Indeed, replacing CH_2_ with S causes the dihedral angle between the lone pair of O and the axis of the aromatic π system to increase from 17° in tocopherol to about 30° in the 4-thiachromanol structure (see Scheme 4), decreasing the ED contribution of O [46]. Optimization of the 4-thiaflavane structure brought to compounds **10** and **11**, in which the chromanol OH was shifted in *ortho* to the sulfide [47] (Scheme 4). The geometrical constraints allow only the onsetting of very weak intramolecular H-bond of the OH to the neighboring sulphur, while forcing its ED contribution, which sums up to that of the MeO substituent inserted to compensate the diminished conjugation with the endocyclic oxygen. As a result, both **10** and **11** were better antioxidant than reference α-tocopherol: both had higher *k*_inh_ (4.0 × 10^6^ and 3.4 × 10^6^ M^−1^s^−1^, respectively) and **9** was able to trap twice as many ROO• radicals (*n* = 4 vs. 2) as α-TOH, owing to the presence of the catechol function. Aiming at further improving the stereo-electronics, the fused 6-membered ring in the 5-hydroxy-1,4-benzo[b]oxathiine core was shrunk to a 5-membered ring by removal of the endocyclic oxygen, to afford the 7-hydroxy-2,3-dihydrobenzo[b]thiophene core as in compounds **12** and **13** (Scheme 4) [27], using the same approach that was proven successful by Ingold et al. on passing from the 6-hydroxychromane to 5-hydroxy-2,3-dihydrobenzo[b]furan [48]. Opposite to expectations, owing to the longer C-S bond compared to the C-O bond, the dihedral angle increased slightly in compounds **12**, **13** compared to **10**, **11**, causing a decrease in the reactivity with ROO• radicals (*k*_inh_ ≈ 1.5 × 10^5^ M^−1^s^−1^ at 303 K in chlorobenzene). Aromatization of the 5-membered ring to afford a benzothiophene core was found to better stabilize the phenoxyl radical, due to the onsetting of a sulphur-oxygen non-covalent dipolar interaction. This feature was, therefore, combined with the optimized 5-hydroxy-1,4-benzo[b]oxathiine core to afford compound **14**, which also featured the phytyl chain to increase lipophilicity and better mimic α-tocopherol [29] (Scheme 4). As a result, **14** was three-fold more effective than native α-tocopherol in trapping ROO• radicals and was able to bind to tocopherol transfer protein (TPP) with similar affinity as all-rac. α-tocopherol, promising improved biological activity [29].

**Scheme 5:**
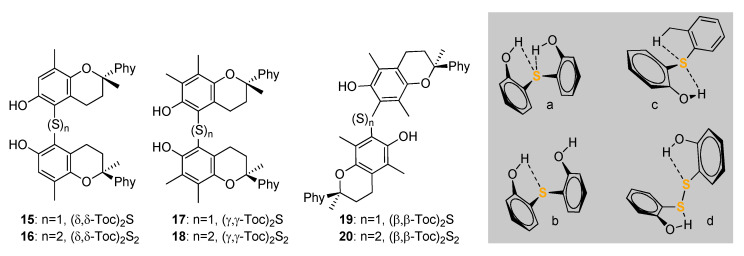
Ditocopherols featuring -S- and -S-S- functions, and representation of the possibilities for intramolecular H-bonding of sulfides (**a**–**c**) and difulfides (**d**).

A recent development in the chemistry of S containing tocopherols is represented by the thia-bridged δ-,γ- and β-ditocopherols, (Toc)_2_S, **15**, **17**, **19** and the corresponding ditocopheryl disulfides (Toc)_2_S_2_ **16**, **18**, **20**, along with the full range of unsymmetrical ditocopheryl sulfides [49] (Scheme 5). Interestingly, these derivatives have redox properties and antioxidant activity which are largely dictated by the occurrence and strength of intramolecular H-bonding of the phenolic OH to the sulfide and disulfide bridge. While all compounds have good antioxidant activity, the reaction with ROO• radicals is about 10-fold faster for sulfides compared to disulfides, owing to the difference in H-bonding to sulphur, which was found to be ca. 4 kcal/mol stronger in disulfides [49].

**Scheme 6:**
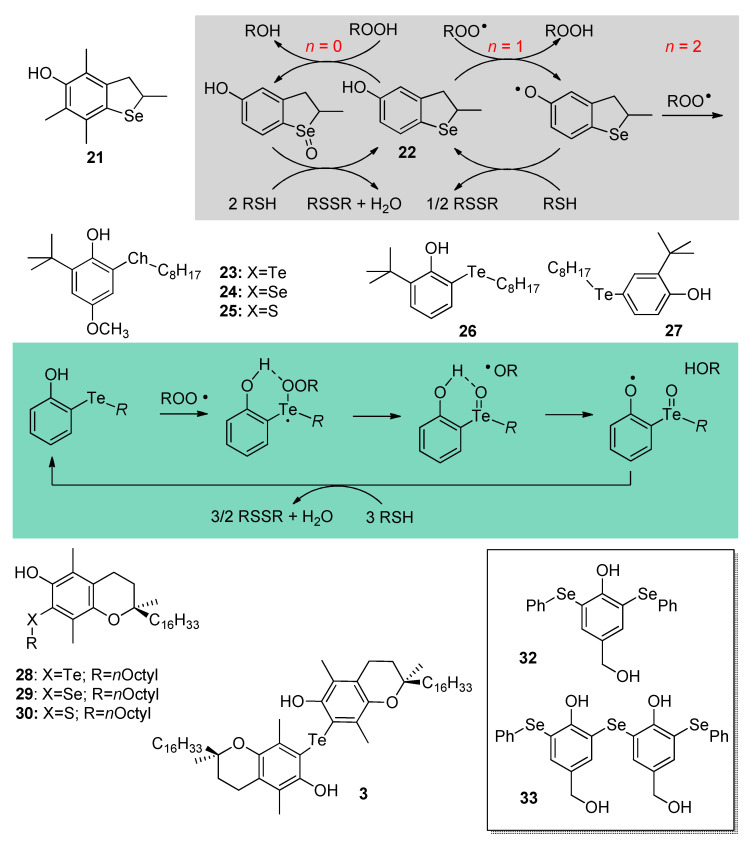
Se- and Te-containing antioxidants endowed with catalytic or additional mechanisms of antioxidant activity.

Perhaps the most striking modification of the antioxidant activity of biomimetic phenols was achieved upon substitution with heavier chalcogens. Focusing on Se, the search for antioxidants with improved stereo-electronics over selenotocopherol **3** afforded a family of 2,3-dihydrobenzo[b]selenophene-5-ol antioxidants [50]. Among them, compound **21** (Scheme 6) having similar substitution pattern on the aromatic ring as compound **3** (Scheme 4), showed slightly lower BDE (OH) (77.6 kcal/mol vs. 78.1 kcal/mol) and slightly higher reactivity with peroxyl radicals in lipophilic solution (*k*_inh_ = 1.5 × 10^6^ M^−1^s^−1^ at 303 K in chlorobenzene). When tested in a stirred water/cholorobenzene biphasic system, it protected linoleic acid similarly to α-tocopherol, although the duration of protection was slightly shorter (i.e., it apparently trapped less than *n* = 2 peroxyl radicals), due to oxidation of the selenide function to the corresponding selenoxide (>Se → >Se=O), by air or by hydroperoxides (ROOH) always present in trace amounts in linoleic acid, which increases the BDE (OH) due to its EW character (see Section 2) [50]. The least substituted **22** had higher BDE (OH) and lower ROO• trapping reactivity in organic solution (BDE = 81.6 kcal/mol; *k*_inh_ = 3.8 × 10^5^ M^−1^s^−1^); however, it showed a surprising reactivity in the biphasic system (Scheme 6).

When 1 mM *N*-acetylcysteine (NAC) was added to the aqueous phase the duration of the antioxidant protection given by **22** was extended about 6-fold, due to regeneration by the thiol, which acted as sacrificial reducing agent, being progressively consumed with time. The duration of the inhibition was proportional to the initial concentration of NAC in the water phase proving the onsetting of a complex catalytic antioxidant behavior by the antioxidant **22** as exemplified in Scheme 6. It is noteworthy that neither compound **21**, nor selenotocopherol **3**, nor even native Vitamin E can be regenerated by thiols; hence, the antioxidant behavior of **22** represents a breakthrough in antioxidants’ design.

Aiming at identifying the minimum structural requirement for regenerability by thiols, the simpler phenols **23**, **24**, **25**, analogues of the known antioxidant BHA bearing, respectively, -TeR, -SeR, or -SR substituents in *ortho* to the OH, were investigated [51] (Scheme 6). All had BDE (OH) values similar to BHA lacking the -ChR substituent (respectively, 78.9, 79.8, 80.6 kcal/mol vs. 80.3 kcal/mol) with modest antioxidant activity due to an intramolecular H-bond to the chalcogen which counterbalanced the ED contribution of the substituent (see Section 2), except Te-substituted compound **23** that trapped ROO• radicals over two-orders of magnitude faster than expected on the basis of its BDE (OH), outperforming α-TOH by over 3-fold both in chlorobenzene solution (*k*_inh_ = 1.0 × 10^7^ M^−1^s^−1^ at 303K) and in biphasic medium. Compound **23** but not **24** and **25** could be regenerated by thiols both in organic solution and in the biphasic system, showing a clear advantage of substitution with Te over Se and S. Additionally, **23** has Glutathione Peroxidase (GPx) mimic antioxidant activity, being able to decompose H_2_O_2_ about as efficiently as synthetic mimic ebselen [51]. Further studies revealed that even simpler phenols bearing the Te-alkyl function in *ortho* position, such as **26**, possessed identical reactivity and antioxidant behavior, irrespective of the lack of other ED substituents in the ring, and, counterintuitively, they were clearly more efficient than the corresponding *para* isomers such as **27** (Scheme 6). It was shown that they react via a different, unique mechanism consisting in O-atom transfer from ROO• radicals to Te followed by (in cage) H-atom transfer to the newly formed RO• radical. Sacrificial reduction by thiols regenerates the antioxidant, which acts in a catalytic fashion (Scheme 6) [52]. This fascinating antioxidant chemistry was exploited in a series of Ch-substituted tocopherol derivatives including compounds **28**–**30**, again proving the superior performance of Te-substitution in *ortho* over that with lighter chalcogens (Scheme 6). Compound **28** matched α-TOH antioxidant activity in the biphasic system in the absence of NAC and largely outperformed it upon addition of NAC [53]. Following the same line, tellurobistocopherol **31** trapped ROO• 12-fold faster than α-TOH in the presence of NAC, with 6-fold longer antioxidant protection due to regeneration by the thiol [54].

It should be pointed out that, while some Se- and most Te-substituted phenolics **22**–**31** have excellent antioxidant activity and can be regenerated by thiols, the efficiency of such regeneration is modest and, typically, 4–8 equivalents of thiols are consumed to regenerate 1 eq. of phenol. This implies that the same compounds can also display marked pro-oxidant activity in biological systems, acting as GSH-depleting agents [52]. This, however, can appropriately be exploited in chemotherapy approaches based on the different oxidative status of healthy vs. cancer or microbial cells, in which they can serve as redox-active selectively cytotoxic drugs [55]. Indeed, redox medicine based on Se and Te organochalcogens is a promising approach for multi-factorial diseases [56]. The use of organoselenium compounds as inhibitors of specific thiol-containing enzymes also contributes to their therapeutical potential [57].

Not surprisingly, the search for novel Ch-functionalized phenolics keeps attracting major interest and offering unconventional antioxidants such as bis (*ortho*-organoselenium) phenols **32** and **33**, which were found to quench radicals with similar efficiency as α-TOH, while boosting a GPx-like antioxidant behavior similar to ebselen [49]. Beside tocopherols and flavanols, other natural phenols offer interesting core structures to explore the role of Ch-functionalization.

The case of the pyridoxine system that was modified to incorporate selenium in various oxidation states in place of the methyl group in position 2 is representative. Some of these organoselenium compounds showed marked GPx-like activity up to 2-fold higher than ebselen [58]. Along this line, insertion of -TeR ring substituents in the 3-pyridinol core afforded extremely potent catalytic chain-breaking antioxidants, capable of trapping peroxyl radicals up to 6-fold more efficiently than α-TOH, being also regenerable by thiols, and boosting a GPx-like activity up to 25-fold higher than that of reference diphenyldiselenide [59].

## 5. Conjugation with Biological Thiols

Thiol conjugation represents a metabolic strategy to reduce the toxicity of exogenous compounds introduced with the diet, by imparting higher water solubility to favor their excretion via the urinary tract.

**Figure 4:**
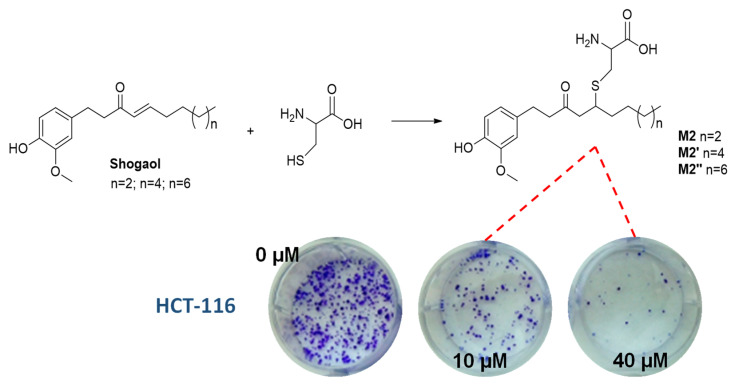
Schematic representation of shogaol conjugates formation (M2) and their ability to inhibit colony formation in human colon cancer cell (HCT-116) with doses ranging from 0 to 40 μM over 2 weeks.

Examples include the phase II mercapturic acid pathway of shoagoals, the major phenolic bioactive components of ginger, which hinge on the addition of GSH to the α,β-unsaturated ketone functional group followed by catabolic hydrolysis to the corresponding cysteine-conjugates. Notably, these conjugates (M2) have proven even more effective than the parent compounds in their activity against human colon and lung cancer cells, while decreasing the toxicity toward normal cells [60] (Figure 4).

As illustrated in the previous paragraph the major effect of introducing a thioalkyl substituent onto a phenol/*ortho*-diphenol scaffold is an enhancement of its antioxidant power.

Several observations have directly or indirectly supported this view. The ability of cysteinyl conjugate of caffeic acid, dihydrocaffeic acid and tyrosol, but not the corresponding catechols, to reduce metmyoglobin to oxymyoglobin clearly indicated a higher reducing potency of the former and suggested their use as preserving agents for retaining the fresh meat color [61].

**Figure 5:**
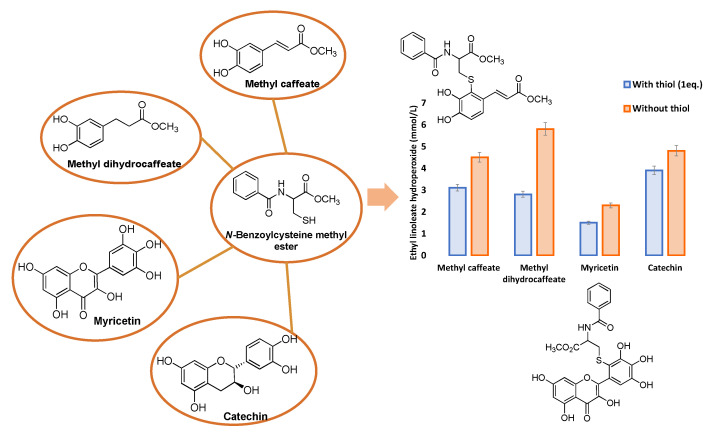
Antioxidant activity of methyl caffeate, methyl dihydrocaffeate, myricetin or catechin with or without equimolar amounts of *N*-benzoylcysteine methyl ester against 2,2′-azobis (2,4-dimethylvaleronitrile)-induced lipid oxidation for 4 h.

Similarly, the enhancement of the antioxidant activity of methyl caffeate and methyl dihydrocaffeate in the presence of excess cysteine as assessed in a lipid oxidation system induced by an azo-compound was ultimately explained in terms of the formation of mono-thiol adduct of methyl caffeate and the mono- and di-thiol adducts of methyl dihydrocaffeate [62] (Figure 5).

A closely related study showed the enhancement of the antioxidant activity of flavonoids, particularly myricetin, in a lipid peroxidation system associated to the presence of the lipophilic cysteine derivative *N*-benzoylcysteine methyl ester. Additionally, in this case analysis of the reaction mixture provided evidence for the presence of two thiol adducts on the B ring, and probably C-ring adducts generated during the oxidation process [63] (Figure 5).

Along this line is also the reported ability of cysteinyl caffeic acid to prevent the laccase mediated oxidation of a typical enzyme substrate such as ferulic acid on account of its higher proneness to oxidation [64].

A noticeable example of the marked effects of the conjugation of catechols with thiols on the reactivity of the final adducts is offered by cysteinyldopas, a group of metabolites that are biosynthetized in mammalian melanocytes by reaction of cysteine with dopaquinone generated by tyrosinase catalyzed oxidation of tyrosine [65].

**Figure 6:**
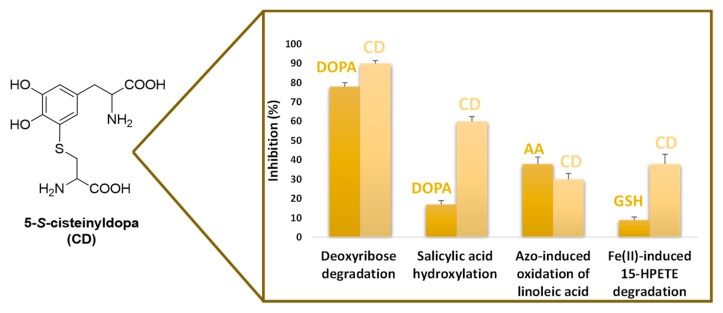
Effect of 5-*S*-cysteinyldopa (CD) on: deoxyribose degradation and salicylic acid hydroxylation induced by the Fenton reagent; conjugated diene formation in the 2,2′-azobis (2-amidinopropane) (ABAP)-induced oxidation of linoleic acid and malondialdehyde formation from Fe^2+^-induced 15-HPETE degradation. For comparative purposes the effects of DOPA, ascorbic acid (AA) and GSH were examined at the same concentration.

The major adduct formed in the metabolic pathway, 5-*S*-cysteinyldopa (CD), occurring in body fluids urine and serum at nano to micromolar levels, proved to be a potent inhibitor of the Fenton reaction as modelled in the deoxyribose degradation and salicylic acid hydroxylation assays, with effects much higher than those exhibited by DOPA, the corresponding catechol, attributable to the strong binding ability of CD toward iron (III) [66] (Figure 6). CD was also very effective in the inhibition of several models of lipid peroxidation, including the azo-induced oxidation of linoleic acid in SDS micelles, Fe^2+^-induced oxidation of arachidonic acid, malondialdehyde formation from Fe^2+^-induced 15-hydroperoxy-5,8,11,13-eicosatetraenoic acid (15-HPETE) degradation and even thiobarbituric acid reactive substances (TBARs) formation in rat brain cortex homogenates (Figure 6). The effects proved comparable, or in some cases superior, to that of potent physiological antioxidants such as ascorbic acid and GSH [67]. On this basis, the increased production of this circulating metabolite by stimulated melanocytes may be envisaged as part of the biochemical machinery that is set into motion to counteract threatening events consequent to oxidative stress, including melanoma [66,68]. Along this line of interpretations are the increased plasma levels of CD observed in patient with psoriasis, a pathological condition associated to oxidative stress conditions, as evidenced by the concomitant raise of lipid peroxidation markers TBARs [69].

The major product of conjugation of cysteine with dopamine, 5-*S*-cysteinyldopamine, is commonly regarded as the main precursor of neuromelanin, the pigment of *substantia nigra* in the human brain playing a critical role in Parkinson’s disease [70]. Formation of the 2- and 5-*S*-cysteinyl dopamine adducts under oxidative conditions mimicking those occurring in the disease, e.g., in the presence of reactive nitrogen species such as peroxynitrite, may result in the exacerbation of the neural injury due to the higher reactivity of the adducts leading to oxidation to neurotoxic species, likely benzothiazine and pigment thereof, as extensively described in the case of CD [71,72,73].

Besides the role played in the onsetting and progression of Parkinson’s disease, the pigment formed by oxidative polymerization of cysteinyldopamine has attracted interest from the application viewpoint because of its ability to form films on almost any substrate, similarly to what was reported for polydopamine (PDA), a reference adhesive material derived from dopamine oxidation [74,75,76].

**Figure 7:**
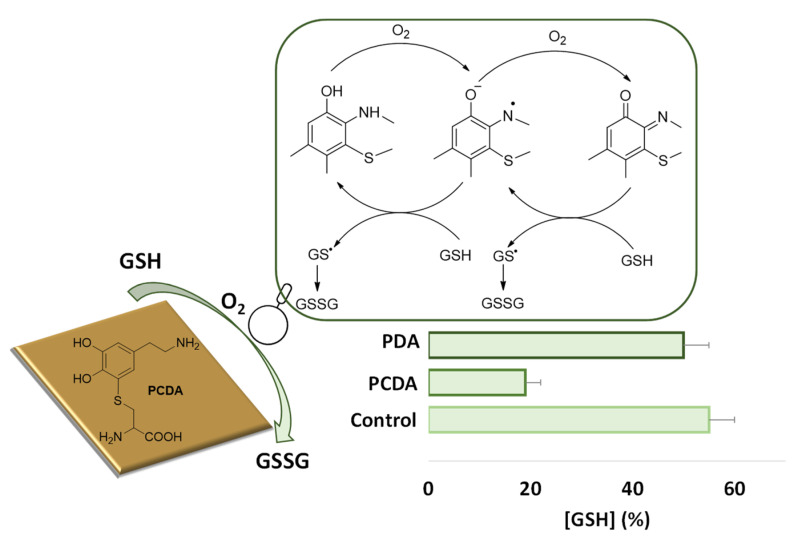
Effect of polycysteinyldopamine (PCDA) or polydopamine (PDA) thin films on the autoxidation of GSH after 3 h incubation in phosphate buffer pH 7.4. The redox cycling mechanism supposedly involved in PCDA prooxidant properties toward GSH is also shown.

Additional interesting properties exhibited by these films include a marked redox responsiveness. Films obtained by aerial oxidation of 5-*S*-cysteinyldopamine proved able to accelerate GSH autooxidation, whereas those prepared from dopamine under the same conditions were inactive. These effects were attributed to the redox cycling properties of benzothiazine units present in the film, as a result of cysteinyldopamine oxidative polymerization (Figure 7) [77].

In a related study, polymers obtained by co-oxidation of dopamine and 5-*S*-cysteinyldopamine showed photoresponsive properties to an extent that was proportional to the content of 5-*S*-cysteinyldopamine. These polymers exhibited an increasing absorption in the visible region with increasing 5-*S*-cysteinyldopamine content, and this feature could account for the marked photocapacitative response in metal-insulator semiconductor (MIS) devices on visible light irradiation [78].

**Figure 8:**
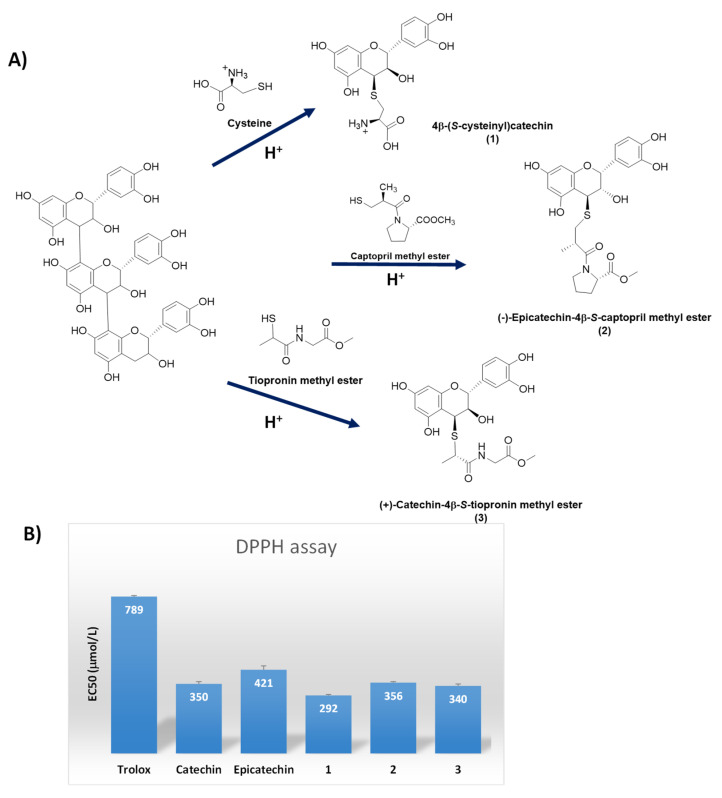
(**A**) Schematic overview of the flavan-3-ol thio-conjugates (1–3) formation by depolymerization of grape polymeric flavanols in the presence of cysteine, captopril or tiopronin under acidic conditions; (**B**) Free radical scavenging activity of the thio-conjugates in the DPPH• assay. The scavenging ability of Trolox, catechin and epicatechin is also reported for comparison.

Thiol-induced depolymerization of catechin polymers in grape seeds proved a straightfoward route for the preparation of a class of biobased thiol conjugated compounds endowed with antioxidant activity higher than the starting catechins. In addition to cysteine, another two bioactive thiol compounds were used, i.e., captopril and tiopronin, leading to a series of derivatives that could be obtained in pure form by high-speed counter-current chromatography (HSCCC) and semi-preparative HPLC fractionation [79] (Figure 8).

The 4-*S*-cysteinyl conjugates of epicatechin, catechin, and epicatechin 3-*O*-gallate were similarly obtained by cysteine induced depolymerization of grape procyanidins [80]. The adducts proved more efficient than the underivatized epicatechin as scavengers of the DPPH• radical (Figure 8) and weak growth inhibitors of human colon carcinoma HT29 cells [80].

In a similar approach, conjugation of epicatechin and epicatechin 3-*O*-gallate with cysteine and cysteine derivatives led to compounds with enhanced neuroprotective activity compared to underivatized epicatechins as evaluated on HT-22 nerve cells death induced by exposure to glutamate. The mechanisms underlying these effects involved scavenging of ROS generated in mitochondria, a process that was minimal or null for the underivatized epicatechin [81]. All the conjugates were able to scavenge mitochondrially generated ROS inside the cells.

**Scheme 7:**
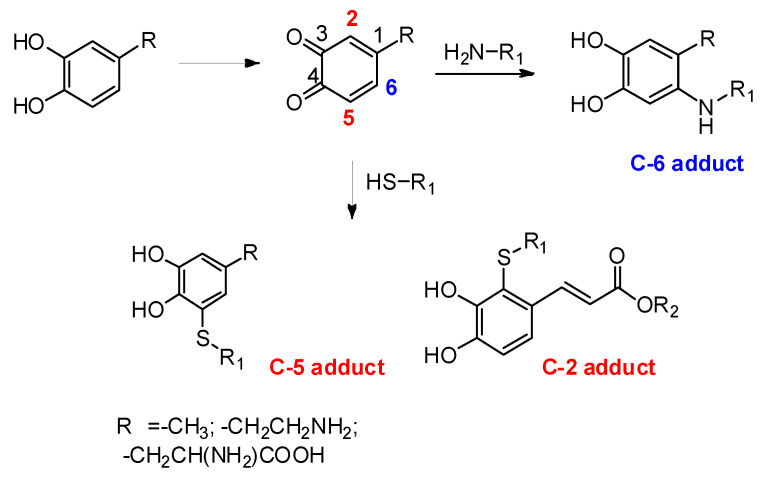
Schematic overview of the regiochemistry of thiol/*ortho*-quinone reactions.

Of particular interest is also the regiochemistry of thiol/*ortho*-quinone reaction (Scheme 7). Differently from the usual 1,4-nucleophilic addition to the *ortho*-quinone that is observed with all other nucleophiles, e.g., amines, leading to C-6 conjugates, the coupling reaction of thiols displays an anomalous 1,6-type regiochemistry leading mainly to C-5-linked adducts as observed in the case of dopamine and DOPA (Scheme 7). This is an intriguing, still unresolved, mechanistic issue that awaits for a detailed and convincing interpretation.

Replacement of the alkyl chain of catecholamines with electron withdrawing substituents directs the attack of the thiol toward the more hindered 2-position of the *ortho*-quinone ring [82,83,84] (Scheme 7).

Representative examples are hydroxycinnamic acids such as caffeic acid and derivatives found in natural sources such as chlorogenic acid and caftaric acid, for which 2-*S* adduct were obtained by oxidation with polyphenoloxidase in the presence of GSH, cysteinylglycine or cysteine [85,86,87].

GSH, which plays a key role in the detoxification processes, imparts water solubility and hence appears an obvious choice for the preparation of derivatives of polyphenols of dietary interest, with improved antioxidant properties for use, e.g., as additives for preserving food during storage. Another important role that antioxidant dietary polyphenols may play is to act as chemopreventive agents, controlling tumor-initiating events in the gastrointestinal tract by the scavenging of the reactive nitrogen species (RNS) that are generated at the acidic pHs of the gastric compartments following intake of food containing nitrite ions up to micromolar levels, e.g., cured meats, vegetables, etc. Nitrosation, nitration and oxidation of crucial biomolecules brought about by RNS may ultimately cause toxic, mutagenic, and carcinogenic effects [88,89].

**Figure 9:**
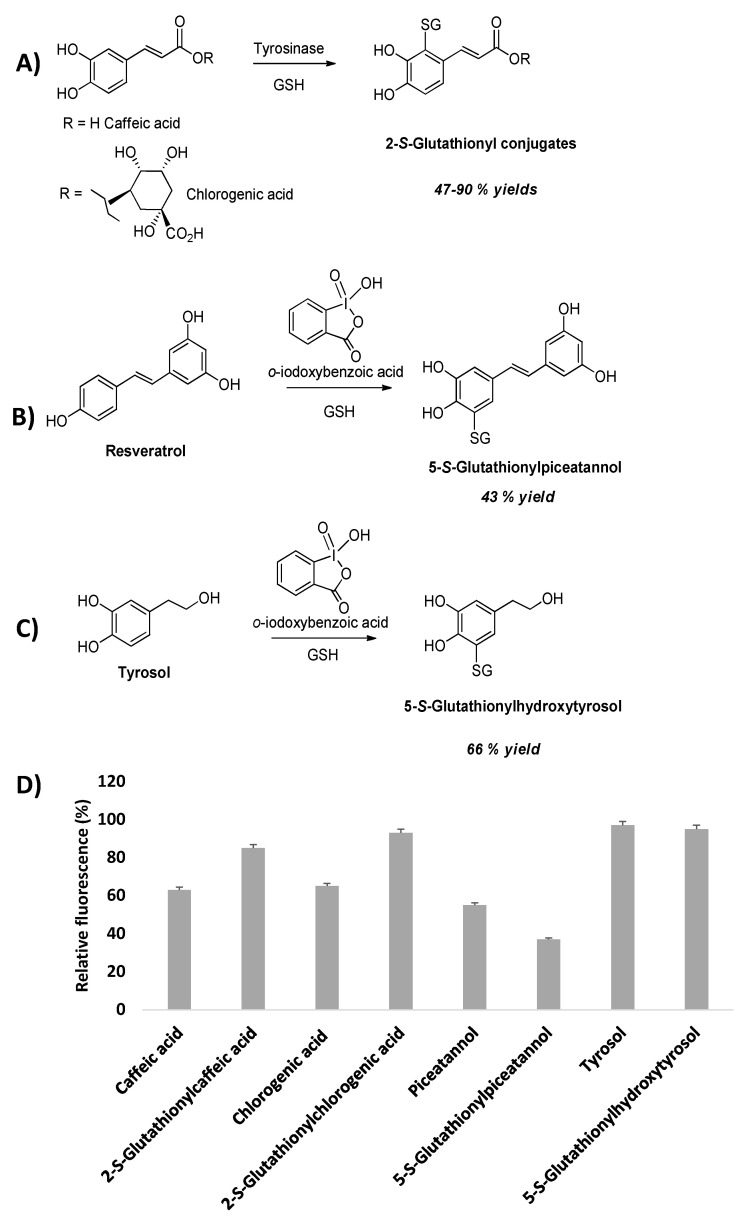
Schematic overview of the glutathionyl adduct formation (**A**–**C**) and (**D**) effects of catechols and their glutathionyl conjugates on DAN nitrosation at pH 4.0 measured as fluorescence emission at 450 nm of naphtho [2,3-d]triazole.

The 2-*S*-glutathionyl derivatives of caffeic and chlorogenic acid could be prepared in substantial yields by nucleophilic addition of GSH to the *ortho*-quinones generated by tyrosinase oxidation (Figure 9). Moreover, glutathionylpiceatannol obtained by regioselective *ortho*-hydroxylation and oxidation of resveratrol using the *ortho*-iodoxybenzoic acid method [90] followed by conjugation with GSH led to the 5-*S*- adduct due to the lack of the electron-withdrawing effect of the substituent of the *ortho*-quinone ring, e.g., the carboxyl group in cinnamic acids derivatives [85,86,91] (Figure 9). Application of the *ortho*-iodoxybenzoic acid method on tyrosol followed by GSH addition to the in situ generated *ortho*-quinone of hydroxytyrosol allowed access to the 5-*S*-glutathionyl adduct (Figure 9).

The *S*-glutathionylpiceatannol was found to be one of the most effective inhibitors in the diaminonaphthalene (DAN) nitrosation assay, even more potent than the parent piceatannol that had been proposed as a new reference compound in the field, replacing the originally used caffeic acid [91]. Intriguingly, sulphur conjugation with the caffeic acid system resulted in an opposite effect on the inhibition activity. Indeed, in the case of *S*-glutathionylpiceatannol a stabilizing effect of the GSH moiety on the phenoxyl radical intermediate, crucial to the inhibition mechanism, may be invoked, whereas the same group may reduce the reactivity of the propenoate chain of glutathionylcaffeic acid relative to caffeic acid itself by steric effects, forcing the side chain double bond out of planarity with the catechol moiety, thus hindering the electron delocalization over the phenylpropenoate pi-framework.

The trend observed in the antioxidant DPPH• assay was completely different, suggesting that hydrogen donor efficiency is not associated to the antinitrosating activity that rather requires ability to react with RNS such as N_2_O_3_ generated in the DAN assay.

A prominent biological thiol with a marked lipophilic character is dihydrolipoic acid. This thiol, easily obtained by reduction of lipoic acid under mild conditions, exhibits structural features that can be exploited in the design of catechol systems active in different biological compartments. Indeed, in addition to the free primary SH group that allows for conjugation with the catechol system powering its hydrogen donor ability, the flexible octanoic acid chain imparts to the conjugate, the ability of interacting with cellular membranes, while the second SH group remains free for further derivatization.

A number of conjugates of dihydrolipoic acid with catechols have been prepared and the antioxidant power was evaluated together with other biological activities.

**Figure 10:**
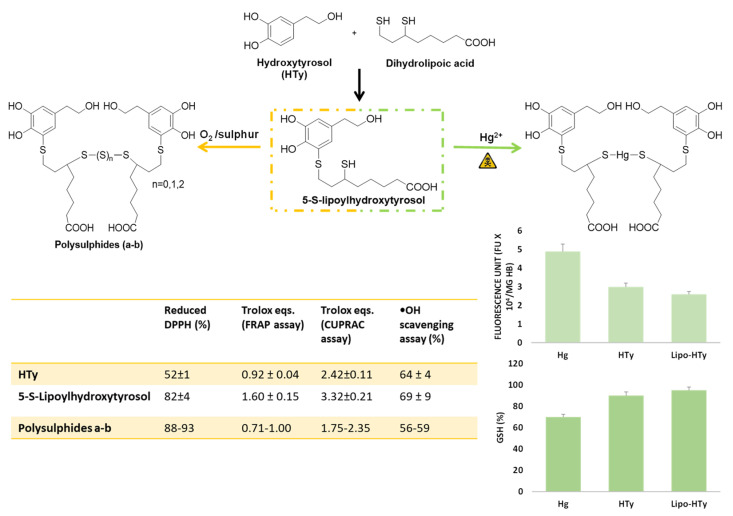
Synthesis and evaluation of the antioxidant activity of 5-*S*-lipoylhydroxytyrosol and its derivatives with sulphide chain. Cytoprotective effects of Lipo-HT and HTy on Hg-induced oxidative alterations in red blood cells.

Notably, 5-*S*-Lipoylhydroxytyrosol could be obtained by facile regioselective reaction of dihydrolipoic acid with hydroxytyrosol *ortho*-quinone using the same protocol developed for preparation of the GSH conjugate (Figure 10). It spontaneously oxidizes to the disulfide upon standing in mildly alkaline (pH 7.4) aqueous solution in the presence of oxygen, whereas, following the addition of sulphur, it can be converted into the tri- and tetra-sulfide depending on the reaction medium [92] (Figure 10). The polysulphides thus obtained are of particular interest as they combine in their structure the thioalkyl catechol moiety, expectedly endowed with high antioxidant activity, with the polysulphide bond typically associated to a variety of chemical, biological and pharmacological properties, as observed in the case of garlic polysulfides [93], which are known to act as ROS scavengers and antiproliferative agents [94].

All the lipoylhydroxytyrosol derivatives exhibited a stronger hydrogen donor ability than the reference compound Vitamin E analogue Trolox in the DPPH• assay (88−93% reduction vs. 68% by Trolox), and, in particular, 5-*S*-lipoylhydroxytyrosol proved a more potent antioxidant than hydroxytyrosol, as observed for other *S*-conjugates with respect to the parent catechols, including 5-*S*-glutathionylhydroxytyrosol, as discussed above (Figure 10).

A marked activity was observed for the dihydrolipoyl acid conjugates in the ferric and copper-reducing-antioxidant power (FRAP and CUPRAC) assays, with 5-*S*-lipoylhydroxytyrosol exhibiting the highest efficacy also compared to hydroxytyrosol (Figure 10), although, in addition to the effect of thioalkyl substitution, the contribution to the overall reducing activity provided by the free secondary SH group should be duly considered. Conversely, the trend observed with the polysulphides that showed a decrease in the iron reducing power with increasing the number of sulphur atoms may possibly be ascribed to a progressive decrease in the solubility in water that is the medium in which the FRAP and CUPRAC assays are run. Similarly, 5-*S*-lipoylhydroxytyrosol proved to be a highly active hydroxyl radical scavenger at concentration as low as 10 μM, as determined by salicylic acid hydroxylation inhibition assay, whereas all the polysulphides showed a lower and comparable potency [92] (Figure 10).

Other model systems were used to evaluate the antioxidant activity of the dihydrolipoic acid conjugates. The effects of the compounds on lipid peroxidation were measured by determining the kinetics of the reaction with peroxyl radicals under different conditions. In polar solvents such as acetonitrile, the reactivity of hydroxytyrosol was significantly lowered since the reactive OH group was engaged in H-bonding with the solvent. However, in the conjugate 5-*S*-lipoylhydroxytyrosol this effect was markedly less pronounced because of the neighboring SH moiety “protecting” the OH group from interaction with the solvent. As a result, the conjugate proved a very effective antioxidant compared to common reference compounds, either natural such as quercetin or synthetic such as 3,5-di-*tert*-butylcatechol. Determination of the BDE of the 4-OH group in *tert*-butanol by EPR methodologies consistently indicated a ca. 1.5 kcal/mol lower value for 5-*S*-lipoylhydroxytyrosol relative to hydroxytyrosol. This result was also confirmed by DFT calculations [33].

In addition to chemical assays, the activity of both 5-*S*-lipoylhydroxytyrosol and the polysulphides in a validated cellular system was determined using a range of concentrations (1–20 µM) close to the levels of hydroxytyrosol found in the plasma of individuals who consume extra virgin olive oil in Mediterranean countries. The compounds proved non-toxic and, in addition, exhibited marked protective effects against ROS generation, showing inhibition values in the range 60−85% at 1 μM concentration, as well as toward cell damage induced by *t*-BHP, to higher extent with respect to hydroxytyrosol [92].

Notably, 5-*S*-lipoylhydroxytyrosol exhibited a protective effect also in another cellular model system, that is oxidative stress induced on human erythrocytes by treatment with mercury salts. At concentrations 5–20 μM, the compound decreased the Hg-induced generation of ROS, hemolysis, and the depletion of intracellular GSH levels to a higher extent with respect to hydroxytyrosol [95] (Figure 10).

Other dihydrolipoic acid catechol conjugates have been prepared and tested for their activity. The caffeic acid-dihydrolipoic acid *S*-conjugate, 2-*S*-lipoylcaffeic acid, and its methyl ester proved effective inhibitors of mushroom tyrosinase catalyzed oxidation of tyrosine or DOPA to melanin pigments. The IC_50_ values of 3.22 ± 0.02 µM (0.05 ± 0.01 µM for the methyl ester) for the catecholase activity of the enzyme and of 2.0 ± 0.1 µM (0.83 ± 0.09 µM for the methyl ester) for the cresolase activity, indicated a greater efficiency of these compounds compared to the parent caffeic acid (and the methyl ester) and the standard inhibitor kojic acid. Most interestingly, a marked inhibitory activity was also found on human tyrosinase from melanoma cells with IC_50_ values for the catecholase activity of 76.2 ± 6.0 µM and 30.1 ± 1.5 µM for lipoylcaffeic acid conjugate and its methyl ester, respectively. Based on these results these conjugates can be regarded as promising depigmenting agents and melanogenesis regulators for the treatment of pigmentary disorders [96,97].

## 6. Conclusions

The examples illustrated in this review have contributed to draw a comprehensive picture of the different effects produced by insertion of sulphur and heavier chalcogens onto the molecular scaffold of phenols. The chalcogen modified phenols may be obtained by introducing substituents containing heavy chalcogens directly onto the aromatic ring, or by isosteric replacement of oxygen with higher chalcogens, and also by conjugation of thiols with catechols. In some cases, complex multistep synthetic procedures are required, as in the case of the many tocopherol analogues, but straightforward preparation protocols have also been developed, as exemplified by the *o*-iodoxybenzoic method to generate labile *ortho*-quinones followed by in situ thiol addition.

Chemical assays of current use have been applied to evaluate the antioxidant potential of the chalcogen compounds with respect to the parent phenols. When available, data on inhibition of lipid peroxidation by trapping alkylperoxyl radicals have been used for discussion, along with other well-known assays such as, DPPH•, ABTS•^+^ and GO• radical scavenging ability, inhibition of the OH• mediated hydroxylation/oxidation of model systems (salicylic acid, deoxyribose), ferric or copper reducing power, scavenging of nitrosating species, or iron binding in the iron-initiated peroxidation.

Of interest in this context are also the mechanistic studies based on experimental and computational investigations that have allowed researchers to detail the mode of action of the different antioxidants, highlighting how this is critically affected by the nature of the medium.

Some of the selenium and tellurium containing phenols proved effective mimics of GPx with an efficiency in decomposing hydrogen peroxide comparable to that of the reference synthetic mimic ebselen.

Most of the examples offered in the review refer to naturally occurring phenols belonging to different classes such as flavonoids, stilbenes, hydroxycinnamic acids, catechol amino acids and catecholamines. In general, it can be concluded that the effects of chalcogens are positive when considered in relation to the ability of the modified phenols to control or inhibit processes that are noxious in biological systems, such as peroxidation of membrane lipids or damage to cellular constituents mediated by reactive oxygen/nitrogen species.

As a final remark, it should be noted that numerous studies in the literature document how chalcogen containing phenols exhibit biological activities that are much lower or not found in the parent systems. This holds for mushroom and mammalian tyrosinase inhibition by 2-*S*-lipoylcaffeic acid and its methyl ester, the neuroprotective activity of epicatechin and epicatechin 3-*O*-gallate conjugates with cysteine and cysteine derivatives, the antitumor activity of shoagoals cysteine conjugates and the oxidative stress protection in intestinal myofibroblasts and osteocytes by benzoselenophene derivatives. Additionally, the pro-oxidant activity of some Se and Te organochalcogens, as well as of cysteinyldopa (mine) oxidation products capable of acting as GSH-depleting agents can be exploited in chemotherapy approaches in which they can serve as redox-active selectively cytotoxic drugs.

Several issues should be addressed in future studies. A more extensive examination of the biocompatibility of the compounds with the highest antioxidant activity in vitro is a high-priority goal with regards to the exploitation of these compounds for therapeutic purposes. Another related issue that deserves much attention concerns the metabolic transformations of heavy chalcogen-modified phenolics in the human organism, in order to assess whether their biological activities are lost, maintained, or even potentiated in the metabolic products.

Moreover, the potential therapeutic value of the organoselenium compounds related to their ability to act as redox active cytotoxic drugs should be further explored in order to guide the design of lead compounds with optimized lifetime and absorption features for use against pathological conditions based on impaired redox balance.
